# On the occasion of the centennial year of the two greatest Croatian soccer teams: brief review of the evidence base for team physicians

**DOI:** 10.3325/cmj.2011.52.1

**Published:** 2011-02

**Authors:** Dario Sambunjak, Jurica Rakić

There is an expression that “soccer is the most important unimportant thing in the world.” Although everyone admits it is “only a game,” enormous importance is attached to it. Social significance of this sport is so great that it inevitably affects the health of people. In one of the most extreme examples, a disagreement at a soccer match sparked a war between two Central American countries, leaving thousands of people dead or displaced (1). But this notorious event is just the tip of the iceberg. Days when soccer games take place – usually Sundays – are associated with a rise in violence, while the soccer fever during great international contests, such as the World Cup or Euro Cup, results in an increased number of emergency calls to ambulance services (2). Fans intuitively know that loud and passionate cheering can influence refereeing decisions in favor of their team (3), but they are perhaps less aware that such behavior is associated with a significant increase in rates of acute cardiovascular events (4,5). Rhetoric of violence is obvious when fans metaphorically demand from players to “die on the soccer field,” but unfortunately, it is not unheard of that players die on the soccer field not only metaphorically but literally (6).

Soccer team physicians have very limited power in preventing public and personal health hazards generated by overenthusiastic soccer aficionados. Their main duty is to keep their players in the best possible health condition to bear the strain of a professional soccer career. Top-tier soccer teams can afford high-profile and expensive technologies to fine-tune their players' physical condition for the optimal performance on the pitch. Teams with smaller budgets can only rely on the knowledge and skills of their physicians to reduce the gap in physical performance that constitutes the difference between successful and less successful teams (7).

Although Croatia has a reputable national soccer team, the Croatian league is small and unimpressive. In terms of money involved, it cannot be compared with the Premier League, Bundesliga, La Liga, or Seria A. Nevertheless, Croatian soccer has a proud history that goes many decades back. This year is the centennial of the two most celebrated Croatian teams – and long-standing rivals – Hajduk from Split and Dinamo from Zagreb. Hajduk, which owes its name to the *hajduks*, romanticized brigands that fought the Ottoman Turks many centuries ago, was officially registered on February 13, 1911 and has played under the same name ever since. Dinamo was founded in the immediate aftermath of the World War II, but is considered the successor of the First Croatian Citizens' Sports Club Građanski, established in Zagreb on April 26, 1911 and disbanded by the communist authorities on June 6, 1945. Dinamo took over not only Građanski's colors, nickname, and fan base, but also most of its players. Thus, both major Croatian soccer teams have a reason to celebrate this year.

As every other professional soccer team, Hajduk and Dinamo have their physicians, who work with players on a daily basis, trying to prevent and treat their common health problems (Table 1). Evidence-based medicine, with systematic reviews of literature on the top of the evidence pyramid, should be of great help in ensuring the most appropriate interventions for the prevention and treatment of injuries in soccer players. A search of the Cochrane Database of Systematic Reviews (CDSR) and Database of Abstracts of Reviews of Effects (DARE), performed on January 26, 2011, using the search term “soccer OR football,” yielded 15 citations. Among them, there were 2 Cochrane systematic reviews – on the prevention of ankle ligament injuries (8) and on the prevention of hamstring injuries (9). Other citations were paper-based systematic reviews indexed in the DARE. Four of these paper-based reviews explored interventions for the prevention of ankle sprains (10-13) and two explored stretching as part of a warm-up for the prevention of exercise-related injury (14,15). Other reviewed the literature on prophylactic braces in the prevention of knee ligament injuries (16), neuromuscular training programs for the prevention of anterior cruciate ligament injury (17,18), protective equipment in preventing concussion (19), mouthguards in sport activities (20), and diagnostics of long-standing groin pain (21). A systematic review by Olsen et al (22) looked specifically at soccer players, focusing on the effectiveness of strategies for preventing injuries, especially in younger participants.

**Table 1:** Common health problems in soccer players, with possible methods of their prevention, treatment and diagnostics used by the health service of the Hajduk soccer team in Split, Croatia

Health problem	Prevention	Treatment	Diagnostics
**Groin pain:** pubic osteitis, abdominal muscle injury, lumbal spine injury, groin pain syndrome (tendon enthesitis of adductor longus and/or abdominal muscles), sport-related hernia and other hernias	good physical preparation, correction of disproportionate strength of abdominal and thigh muscles, treating chronic tendinitis, avoiding abrupt movements	physical rest, physiotherapy, cryotherapy, muscle strengthening and stretching, massage, non-steroidal anti-inflammatory drugs, chiropractic, minimally invasive spine surgery, hernia surgery, cutting of tendons of gracilis and rectus muscles, rehabilitation	physical exam, ultrasonography, magnetic resonance
**Knee injuries:** meniscus tear injury, collateral ligament injury, cruciate ligament injury, rectus femoris tendon rupture, patellar tendon rupture, knee luxation, intra-articular knee fracture, patella fracture, jumpers knee	avoiding cleats, correcting training errors, raising the heel, avoiding hard surfaces	physical rest, immobilization, exercises for quadriceps muscle, physiotherapy, surgery, reposition, extracorporeal shock wave therapy, non-steroidal anti-inflammatory drugs, ultrasound, laser	physical exam, x-ray
**Achilles tendon pain syndrome**	correcting mistakes in training, raising the heel, avoiding hard surfaces	ceasing sport activity, chryotherapy, muscle strengthening and stretching, surgery (excision of degenerative lesions, reconstruction)	ultrasonography, magnetic resonance, x-ray
**Bursitis:** student’s elbow, prepatellar bursitis, Baker cyst, subachromial, retrocalcaneal, trohanteric, septic, blood	avoiding repetition of same movements, brace, wearing adequate equipment (shoes), orthopedic soles	physical rest, chryotherapy, physiotherapy, electrotherapy, magnet therapy, laser, ultrasound, non-steroidal anti-inflammatory drugs, puncture and elastic bandage after puncture, cortisone locally, surgery	physical exam, ultrasonography
**Iliotibial band friction syndrome**	correcting mistakes in training (such as sudden changes in intensity and duration), correcting anatomical variations (eg, difference in leg length)	cryotherapy, stretching exercises, soles, heat therapy	physical exam, ultrasonography, scintigraphy
**Plantar fasciitis**	orthopedic soles, stretching of plantar fascia, achilles tendon, gastrocnemius muscle, soleus muscle, and foot muscles	termination (decrease) of activity, surgical treatment, cryotherapy, ultrasound therapy, galvanic stimulation, friction massage, ice baths and hot-cold therapy, surgery if needed	physical exam, x-ray
**Overuse syndrome**	removing predisposing factors (inadequate shoes, training on hard surfaces and training errors), stretching exercises	termination of activities that led to symptoms, cryotherapy, stretching and strengthening of affected muscle groups, surgery if needed	physical exam, ultrasonography, magnetic resonance
**Sprained ankle**	avoiding hard surfaces, inadequate shoes, training errors	physical rest, immobilization, inserts, bandages, ice, electrical stimulation of muscles, analgesics, physiotherapy	computerized tomography and magnetic resonance if needed
**Muscle sprain**	eliminating bad practice, warming up, cooling	reducing physical activity, ice pack, elastic bandage, lymphatic drainage, analgesics and anti-inflammatory drugs, physiotherapy (electrotherapy, ultrasound- laser-, magnetic-therapy), kinesiotherapy (stretching exercises and “cross friction” massage), extracorporeal shock wave therapy	physical exam, ultrasonography, magnetic resonance
**Medial tibial stress syndrome**	gradual return to training after a reduction of symptoms, orthopedic soles, bandage, sports shoes, warm up, stretching before and after workout	chryotherapy, massage, stretching	physical exam, ultrasonography, x-ray if needed, magnetic resonance
**Stress fractures**	shoes and substrates	physical rest, immobilizing plaster boot with a heel for walking if needed	x-ray, bone scintigraphy
**Sudden cardiac arrest (death)**	identifying predisposing factors, use of automatic external defibrillators, implantable cardioverter defibrillators and antiarrhythmic drugs	emergent defibrillation with automatic external defibrillators (outside the hospital)	physical examination (screening strategies), 12-lead electrocardiography (ventricular tachyarrhythmias, long Q-T, Brugada, Wolf-Parkinson-White and other syndromes); echocardiography (hypertrophic cardiomyopathy, arrhythmogenic right ventricular cardiomyopathy, aortic stenosisand other conditions)

The same keywords (“soccer OR football”) used in a search by PubMed Clinical Queries yielded 84 citations categorized within “Systematic Reviews.” However, a closer look at the titles and abstracts of these articles revealed that only 28 of them were clearly soccer-related systematic reviews, with or without meta-analysis. Out of these, 22 were not indexed either in the CDSR or DARE (23-44).

Of course, this is not an exhaustive list of evidence-based resources to inform the decisions of a soccer team physician, but it is a good place to start. Further relevant findings can be found in other systematic reviews and primary studies published in the CDSR or in specialty journals. Considering the wealth of knowledge stored in the medical literature, soccer team physicians should heed the advice of Střlen et al: “As with other activities, soccer is not a science, but science may help improve performance” (27).
